# Identification of loci associated with susceptibility to Mycobacterium avium subsp. *paratuberculosis* infection in Holstein cattle using combinations of diagnostic tests and imputed whole-genome sequence data

**DOI:** 10.1371/journal.pone.0256091

**Published:** 2021-08-27

**Authors:** Maria Canive, Oscar González-Recio, Almudena Fernández, Patricia Vázquez, Gerard Badia-Bringué, José Luis Lavín, Joseba M. Garrido, Ramón A. Juste, Marta Alonso-Hearn

**Affiliations:** 1 Department of Animal Health, NEIKER- Basque Research and Technology Alliance (BRTA), Derio, Bizkaia, Spain; 2 Doctoral Program in Immunology, Microbiology and Parasitology, Universidad del País Vasco/Euskal Herriko Unibertsitatea (UPV/EHU), Leioa, Bizkaia, Spain; 3 Departamento de Mejora Genética Animal, Instituto Nacional de Investigación y Tecnología Agraria y Alimentaria, CSIC, Madrid, Spain; 4 Departamento de Producción Agraria, Escuela Técnica Superior de Ingeniería Agronómica, Alimentaria y de Biosistemas, Universidad Politécnica de Madrid, Ciudad Universitaria, Madrid, Spain; 5 Doctoral Program in Molecular Biology and Biomedicine, Universidad del País Vasco/Euskal Herriko Unibertsitatea (UPV/EHU), Leioa, Bizkaia, Spain; 6 Department of Applied Mathematics, NEIKER- Basque Research and Technology Alliance (BRTA), Derio, Bizkaia, Spain; University of Illinois College of Veterinary Medicine, UNITED STATES

## Abstract

Bovine paratuberculosis (PTB) is a chronic inflammatory disease caused by *Mycobacterium avium* susbp. *paratuberculosis* (MAP). Genome-wide association studies (GWAS) have identified single nucleotide polymorphisms (SNPs) significantly associated with susceptibility to bovine PTB. The main objective of this study was to identify quantitative trait loci (QTLs) associated with MAP infection in Spanish Holstein cows (N = 983) using combinations of diagnostic tests and imputed whole-genome sequence (WGS) data. The infection status of these animals was defined by three diagnostic methods including ELISA for MAP-antibodies detection, and tissue culture and PCR for MAP detection. The 983 cows included in this study were genotyped with the Bovine MD SNP50 Bead Chip, and the corresponding genotypes were imputed to WGS using the 1,000 Bull genomes reference population. In total, 33.77 million SNP variants per animal were identified across the genome. Linear mixed models were used to calculate the heritability (h^2^) estimates for each diagnostic test and test combinations. Next, we performed a case-control GWAS using the imputed WGS datasets and the phenotypes and combinations of phenotypes with h^2^ estimates > 0.080. After performing the GWAS, the test combinations that showed SNPs with a significant association (P_FDR_ ≤ 0.05), were the ELISA-tissue PCR-tissue culture, ELISA-tissue culture, and ELISA-tissue PCR. A total of twelve quantitative trait loci (QTLs) highly associated with MAP infection status were identified on the *Bos taurus* autosomes (BTA) 4, BTA5, BTA11, BTA12, BTA14, BTA23, BTA24, and BTA28, and some of these QTLs were linked to immune-modulating genes. The identified QTLs on BTA23 spanning from 18.81 to 22.95 Mb of the *Bos taurus* genome overlapped with several QTLs previously found to be associated with PTB susceptibility, bovine tuberculosis susceptibility, and clinical mastitis. The results from this study provide more clues regarding the molecular mechanisms underlying susceptibility to PTB infection in cattle and might be used to develop national genetic evaluations for PTB in Spain.

## Introduction

Paratuberculosis (PTB) or Johne´s disease is a chronic enteritis of domestic and wild ruminants caused by *Mycobacterium avium* susbp. *paratuberculosis* (MAP). PTB is a major problem for animal health and must be notified to the World Organization for Animal Health. In Europe and North America, PTB is considered endemic in dairy cattle, with herd prevalence estimates higher than 50% [1]. This can result in great economic losses to the dairy industry due to decreased milk production, weight loss, replacement cost, reduced slaughter value, a greater risk to other health problems, premature culling or death from the clinical disease, and the costs of veterinary expenses and control measures [2,3]. The most common clinical signs are progressive weight loss, diarrhea, and decreased milk yield [4]. However, most infected animals do not develop clinical disease, and microbiological and immunological diagnostic tests are not sensitive enough to identify them [5]. MAP is also a suspected cause of Crohn´s disease (CD) in humans, making it a potential zoonotic agent [6]. Colorectal cancer is a complication of the two forms of idiopathic inflammatory bowel disease (IBD); colonic CD and ulcerative colitis. Interestingly, MAP bacilli have been detected in the intestines of patients with CD, ulcerative colitis, and IBD-associated colorectal cancer [7,8].

Once a cow shows clinical signs of PTB, there is no effective treatment and the animal is culled. Commercial inactivated vaccines against bovine PTB are very successful in reducing MAP presence in feces and tissues, and in increasing both milk production and cattle productive life in infected farms [9,10]. However, PTB vaccination with heat-killed inactivated vaccines is not allowed in most European countries due to its interference with *Mycobacterium bovis* (Mb) detection tests [11]. Therefore, PTB control is currently based on testing and culling infected animals plus preventing MAP transmission to susceptible animals using voluntary hygiene and management practices [12,13]. PTB infection is commonly detected using Enzyme-Linked Immunosorbent Assay (ELISA) that detects serum and milk antibodies against MAP. Although serum ELISA is a simple, fast, and cost-effective method for diagnosis clinical PTB, it is known to have low sensitivity for MAP-infected animals that do not show clinical signs and could yield erroneous false-negative results [14]. Therefore, repeating testing or complementary methods (culture or PCR) are required to achieve reasonable sensitivity for PTB diagnosis.

Genomic selection could enhance natural resistance to MAP infection and complement existing control strategies [15]. Genetic improvement of disease resistance is a long-term process; however, the results are permanent and cumulative over generations and could result in disease eradication. Previous studies showed heritability (h^2^) estimates of susceptibility to MAP in Friesian cattle ranging from 0.03 to 0.27 [16–18]. Genome-wide association studies (GWAS) using genotyping of SNPs at low density (LD) [19], medium density (MD) [20–26], and high density (HD) [27–29], as well as imputed whole-genome sequences (WGS) [30–32], have identified quantitative trait loci (QTLs) in the *Bos taurus* genome associated with MAP infection. However, few QTLs have been identified with consistent association across studies due to multiple factors including differences in genetic backgrounds and variable disease prevalence in the studied populations, different samples size, different statistical models, differences in sensitivity of the various diagnostic methods used for infection status classification, different definitions for the cases and controls phenotypes, and the low heritable nature of these phenotypes. Given that different populations across the world can have differences in genetic backgrounds, it is entirely reasonable for some genes to be monomorphic in some populations (these would therefore not be associated with PTB in these populations) even when they have a major physiological role in the defense against PTB. Previous GWAS in Holstein cattle defined phenotypes based on ante-mortem tests such as serum ELISA, milk ELISA, MAP fecal culture, or fecal PCR. The ELISA test is most commonly used for the detection of MAP-specific antibodies in infected animals. This test is cost-effective and simple to perform as compared with detection by bacteriological culture. However, the drawback of this method is its low sensitivity for detection during the subclinical phase of infection and could yield false-negative results. Tissue culturing is considered to be the gold standard due to its increased sensitivity and ability to detect viable MAP in subclinical stages of PTB that can be missed with other diagnostic methods [33]. However, tissue PCR and culture are post-mortem tests that can be taken at a single point and are laborious to conduct on a large number of animals.

The first objective of the current study was to calculate the genetic parameters, variance, and h^2^ estimates, for MAP-specific humoral response and/or tissue MAP infection in Spanish Holstein cattle (N = 983) using different diagnostic methods including ELISA for MAP antibodies detection, and culture and PCR for MAP detection in gut tissues. The second objective was to identify genomic regions and candidate genes associated with MAP infection using imputed WGS data and the individual test results or test combinations with h^2^ estimates > 0.08.

## Materials and methods

### Ethics statement

Animals used in this study were not submitted to any *in vivo* experimentation before stunning for slaughter and, therefore, no specific ethics committee authorization was required. The cows were slaughtered in the Bilbao and Donostia municipal slaughterhouses (Basque Country, Spain) under the pertinent Basque (Basque Government Decree 454/1994), Spanish (Spanish Government Law 32/2007 and Royal decree 731/2007), and European (Council Regulation (EC) No 1099/2009) legislation on animal welfare.

### Animals and PTB diagnosis

The Spanish Holstein population included in this study consisted of 983 culled Holstein cattle from several herds located in eight regions: Basque Country (N = 415, 42.21%), Catalonia (N = 220, 22.38%), Navarre (N = 204, 20.75%), Cantabria (N = 70, 7.12%), Aragon (N = 38, 3.87%), Castile and Leon (N = 25, 2.54%), La Rioja (N = 7, 0.71%) and Asturias (N = 4, 0.41%). Only cows (2 years or older, 5.6 years mean age) were included in the analyses as PTB has a long incubation period and older animals are, therefore, more likely to show clinical signs and positive diagnostic results. The cows were slaughtered in two abattoirs located in the Basque country from March 2007 to May 2010. The infectious status of the animals was determined by ELISA for MAP antibodies detection, and tissue culture and PCR for MAP detection [34]. Sampling was systematically performed once a week at the slaughterhouse. In each visit, the first 2 to 6 animals in the line satisfying the breed and age requirements were sampled. Duplicate blood samples were collected from the jugular vein into 10 ml Vacutainer EDTA tubes (BD, Franklin Lakes, NJ, USA) for genotyping and ELISA testing. Since the mucosa from the ileocecal valve-distal ileum and the jejunal caudal lymph nodes are the primary sites of MAP colonization, samples from these tissues were mixed and processed for tissue culture and PCR. Briefly, MAP isolation was performed in duplicate home-made Herrold´s egg yolk (Becton Dickinson, Franklin Lakes, NJ, USA) and Lowenstein-Jensen media (Difco, Detroit, MI, USA), both supplemented with 2 mg/L of mycobactin J (Allied Monitor, Fayette, MO, USA) as previously described [35]. A positive tissue culture result was considered if one or more MAP colonies were observed in any of the four medium slants. Individuals with 0 CFU in all culture samples were considered controls. A second aliquot from the same tissue homogenates was used for DNA isolation and MAP IS900 amplification using a combined extraction and amplification kit (Adiagene, Saint Brieuc, France). PCR amplifications were performed on an ABI Prism 7000 Sequence Detection System (Applied Biosystems, Foster City, CA, US). Samples showing amplification curves with a threshold cycle (Ct) below 40.0 were considered positive. Serum samples were tested for specific antibody production against MAP by using a two-step ELISA paratuberculosis antibody screening and verification kit (IDEXX Laboratories, Inc., Westbrook, ME, USA) according to the manufacturer´s instructions. The results were expressed as optical density values (OD) and categorized as positive according to the sample-to-positive control ratio defined by the manufacturer. Table 1 provides a summary of the number of animals with positive or negative results for each diagnostic test and test combinations. When combinations of diagnostics tests were used, animals were considered cases when they were positive to all the tests, and controls when they were negative to any (+/), or all of them (+/-).

**Table 1:** Number of cows with a positive and negative test result.

Phenotype	Cases (%)	Controls (%)
ELISA (+/-)	70 (7.12)	913 (92.88)
Tissue PCR (+/-)	266 (27.62)	697 (72.38)
Tissue Culture (+/-)	150 (15.26)	833 (84.74)
ELISA-Tissue PCR (+/)	59 (6.00)	924 (94.00)
ELISA-Tissue Culture (+/)	56 (5.70)	927 (94.30)
Tissue PCR-Tissue Culture (+/)	119 (12.11)	864 (87.89)
ELISA-Tissue PCR-Tissue Culture (+/)	55 (5.60)	928 (94.40)
ELISA-Tissue PCR-Tissue Culture (+/-)	55 (7.71)	658 (92.29)

When combinations of diagnostics tests were used, animals were considered cases when they were positive to all the tests and controls when they were negative to any (+/) or all of them (+/-).

### Genotyping and imputation

Peripheral blood samples were taken at slaughter time and DNA was extracted using the QIAmp DNA Blood Mini Kit according to the manufacturer´s instructions (Qiagen, Hilden, Germany). Purified genomic DNA was quantified spectrophotometrically and subsequently genotyped with the Illumina Bovine MD SNP50 Bead Chip at the molecular genetic laboratory service of the Spanish Federation of Holstein Cattle (CONAFE) using the InfiniumTM iScan software for allele assignation (Illumina, San Diego, CA). Individual genotypes were phased using Eagle 2.4 [36] and imputed with minimac4 [37] to the Bovine HD Bead Chip using a reference panel of 1,278 *Bos taurus* from Run7.0 of the 1,000 Bull Genomes project and 581,712 SNPs (ASR-UCD1.2). Imputation to the WGS level was then undertaken using a reference population of 2,333 *Bos taurus* from Run6.0 of the 1,000 Bull Genomes project [38]. In total, 33.77 million SNPs per animal were identified across the genome. All the SNPs passed a call rate > 0.80. PTB-associated SNPs with minimum allele frequency (MAF) < 0.01 were removed. The number of SNPs kept in the analysis was 13,881,067.

### Variance components and h^2^ estimates

The variance components, standard errors (SE), and h^2^ estimates for MAP infection status explained by all the SNPs were calculated using the genome-wide complex trait analysis (GCTA) software 1.93.2, according to the following formula: $h^{2}=\frac{\sigma 2\;G}{\sigma 2\;G+\sigma 2\;e}$ where σ_G_ is the additive genetic effect of the individuals and σ_e_ is the residual variance [39]. The variance components σ_G_ and σ_e_ in the equation were estimated by the genomic-relatedness-based restricted maximum-likelihood (GREML) approach implemented in GCTA. This approach estimates the proportion of phenotypic variation that can be explained by all genome-wide SNPs using an SNP-derived genetic relationship matrix. The concept behind this method is to fit all the SNPs simultaneously as random effects in a mixed linear model to estimate the variance explained by all the SNPs. GCTA implements the method in two steps; first generating the GRM between individuals and then estimating the variance explained by all SNPs by a Restricted Maximum Likelihood (REML) analysis of the phenotypes with the GRM.

### Genome-wide association study (GWAS)

Imputed genotypes and the results from the diagnostic tests with h^2^ > 0.08 were analyzed in a case-control study using the *mlma* (mixed linear model) association analysis of the GCTA 1.93.2 which fits the effects of all the SNPs as random effects. The model is *y = a + bx + g + e*, where *y* is the phenotype, *a* is the mean term, *b* is the additive effect (fixed effect) of the candidate SNP to be tested for association, *x* is the SNP genotype indicator variable coded as 0, 1 or 2, *g* is the polygenic effect (random effect) i.e. the accumulated effect of all SNPs (as captured by the GRM calculated using all SNPs), and *e* is the residual [39]. Age was included as a covariate in the analysis. After the GWAS, all the SNPs had R^2^ values higher than 50% and were retained, with a mean R^2^ close to 90%. SNPs with R^2^ between 50 and 70% were scarce. To account for multiple testing, a 5% chromosome-wise false discovery rate (P_FDR_ ≤ 0.05) was used to determine the probability that associations were not false positives. Uncorrected P-values between P = 5 × 10^−5^ and P = 5 × 10^−7^ provided a moderate significance level (α), and uncorrected P-values < 5 × 10^−7^ were used to identify highly significant associations (Wellcome Trust Case Control Consortium, 2007). The inflation factor (λ) and quantile-quantile plots were calculated to compare observed distributions of–log (P-values) to the expected distribution under the no association model for each phenotype. λ value close to 1 suggests appropriate adjustment for potential substructure and λ > 1.2 suggests population stratification.

The odds ratio (OR) and their 95% confidence intervals (CI) for the SNPs associated with a positive ELISA-tissue PCR-tissue culture diagnosis (P < 5 × 10^−7^) were calculated using logistic regression analysis with the WGassociation function of SNPassoc 1.9.2 under five different genetic models (co-dominant, dominant, recessive, over-dominant, and log-additive) [40]. The WGassociation function fits individual logistic regression models to each of the class variables (genotypes). For each SNP and genetic model, the function WGstats of SNPassoc 1.9.2. provides genotype frequencies, OR, and 95% CI with the major homozygous genotype deemed as the baseline.

### SNP variants, QTLs, and candidate genes identification

The location of the significant SNPs was determined with biomaRt 2.44.1 for R [41] using the ARS-UCD1.2 reference genome. The genomic distribution of the identified SNPs was determined using the Ensembl Variant Effect Predictor (VEP). QTLs associated with PTB infection were defined based on SNPs on linkage disequilibrium patterns with SNPs that surpassed the suggestive significance threshold (P < 5 × 10^−7^) in a given chromosome. The beginning and end of each quantitative trait locus (QTL) were defined in a window of 500,000 base pairs upstream and downstream by the SNPs that were furthest upstream and downstream of the suggestive SNP. Overlapping QTLs were merged and considered as a single QTL. The defined QTLs were further investigated for the presence of candidate genes within 50,000 base pairs to each side of the defined QTL using Ensembl (https://www.ensembl.org). The identified QTLs and candidate genes were compared with the reported cattle QTLs and genes for PTB susceptibility, bovine tuberculosis (bTB) susceptibility, and clinical mastitis (http://www.animalgenome.org), and with human candidate genes for CD, IBD, and colorectal cancer (https://www.ebi.ac.uk/gwas/). In addition, we uploaded the list of candidate genes and search the Innate DB database for innate immunity genes (https://innatedb.com).

## Results

### Data and descriptive analysis

The infection status of each animal was investigated by serological and microbiological methods. Table 1 presents the descriptive statistics of the analyzed population. Although not included in Table 1, the mean (± SD) OD values were 0.178 ± 0.16 for negative, and 1.882 ± 0.80 for positive animals. ELISA OD values ranged from 0.028 to 3.108. The frequency of ELISA, tissue culture, and tissue PCR positive animals was 7.12, 15.26, and 27.62%, respectively. MAP was detected by tissue culture and PCR in the majority of ELISA-positive animals; 80% and 84%, respectively.

### Variance components and h^2^ estimates

Variance components along with standard errors (SE) and h^2^ estimates were calculated for all the diagnostic tests and their combinations (Table 2). The h^2^ estimates ranged from 0.054 to 0.139 depending on the phenotype. The highest h^2^ estimate was obtained for the ELISA-tissue PCR-tissue culture (h^2^ = 0.139) followed by the tissue culture (h^2^ = 0.101), ELISA-tissue culture (h^2^ = 0.099), tissue PCR-tissue culture (h^2^ = 0.086) and ELISA-tissue PCR (h^2^ = 0.081). When the ELISA was treated as a positive/negative binary trait for MAP status, higher h^2^ estimates were obtained (h^2^ = 0.075) when compared with the ELISA (OD) h^2^ estimates (h^2^ = 0.057).

**Table 2:** Variance components, standard errors (SE), and h^2^ estimates for the diagnostic tests and their combinations.

Phenotype	Additive genetic variance (σ_G_)	SE	Residual variance (σ_e_)	SE	Heritability (h^2^)
ELISA (+/-)	0.004975	0.003905	0.060948	0.004611	0.075464
ELISA (OD)	0.014126	0.013584	0.232861	0.016736	0.057192
Tissue PCR (+/-)	0.010867	0.012009	0.189343	0.014418	0.054277
Tissue Culture (+/-)	0.013107	0.008410	0.115996	0.009433	0.101522
ELISA-Tissue PCR (+/)	0.004584	0.003268	0.051401	0.003860	0.081873
ELISA-Tissue Culture (+/)	0.005307	0.003154	0.047911	0.003653	0.099722
Tissue PCR-Tissue Culture (+/)	0.009149	0.006479	0.096878	0.007505	0.086287
ELISA-Tissue PCR-Tissue Culture (+/)	0.005741	0.003193	0.046567	0.003634	0.109759
ELISA-Tissue PCR-Tissue Culture (+/-)	0.009838	0.005710	0.060478	0.006215	0.139909

OD- Optical density. When combinations of diagnostics tests were used, animals were considered cases when they were positive to all the tests and controls when they were negative to any (+/) or all of them (+/-).

#### GWAS

**Fig 1:**
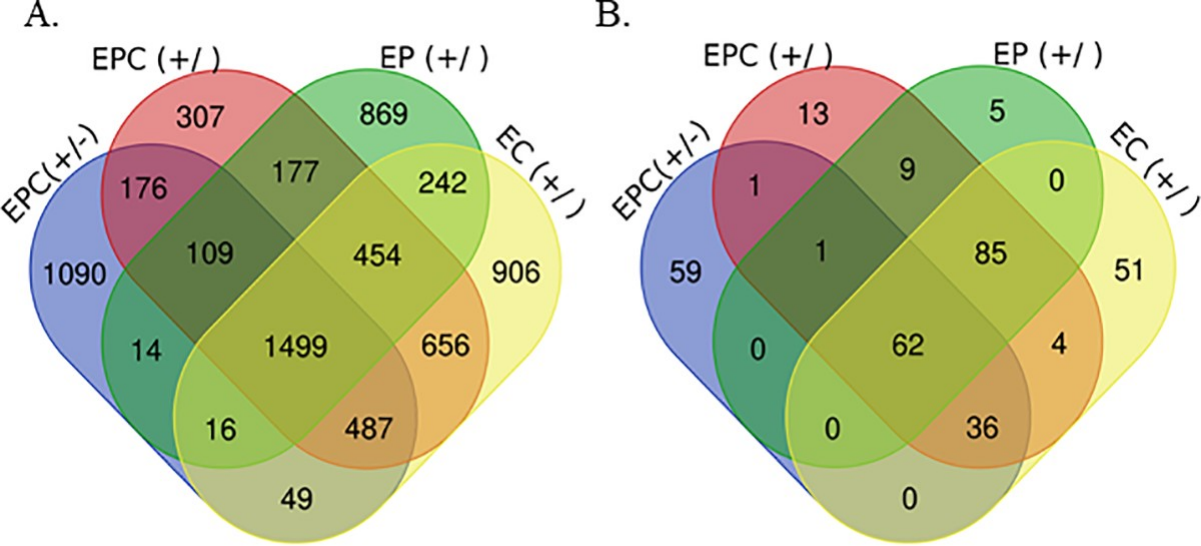
Number of SNPs associated with PTB infection status. The total number of SNPs with moderate (A), or high (B) association with PTB infection status determined by ELISA (E), tissue PCR (P), and tissue culture (C) combinations. Animals were considered cases when they were positive to all the tests, and controls when they were negative to any (+/), or all of them (+/-). A moderate threshold for the association between a SNP and a phenotype was set at a significance level (α) between P = 5 × 10^−5^ and P = 5 × 10^−7^. The high threshold for evidence of an association was set at P < 5 × 10^−7^.

To explore the genetic basis of MAP infection status, a GWAS using the imputed WGS datasets and the phenotypes and combinations of phenotypes with h^2^ > 0.08 was performed. After the GWAS, the phenotypes that showed SNPs with a significant association (P_FDR_ ≤ 0.05) were the ELISA-tissue PCR-tissue culture, ELISA-tissue culture, and ELISA-tissue PCR. The number of SNPs that surpassed the moderate (between P = 5 × 10^−5^ and P = 5 × 10^−7^) and the high (P< 5 × 10^−7^) thresholds are presented in Fig 1.

**Fig 2:**
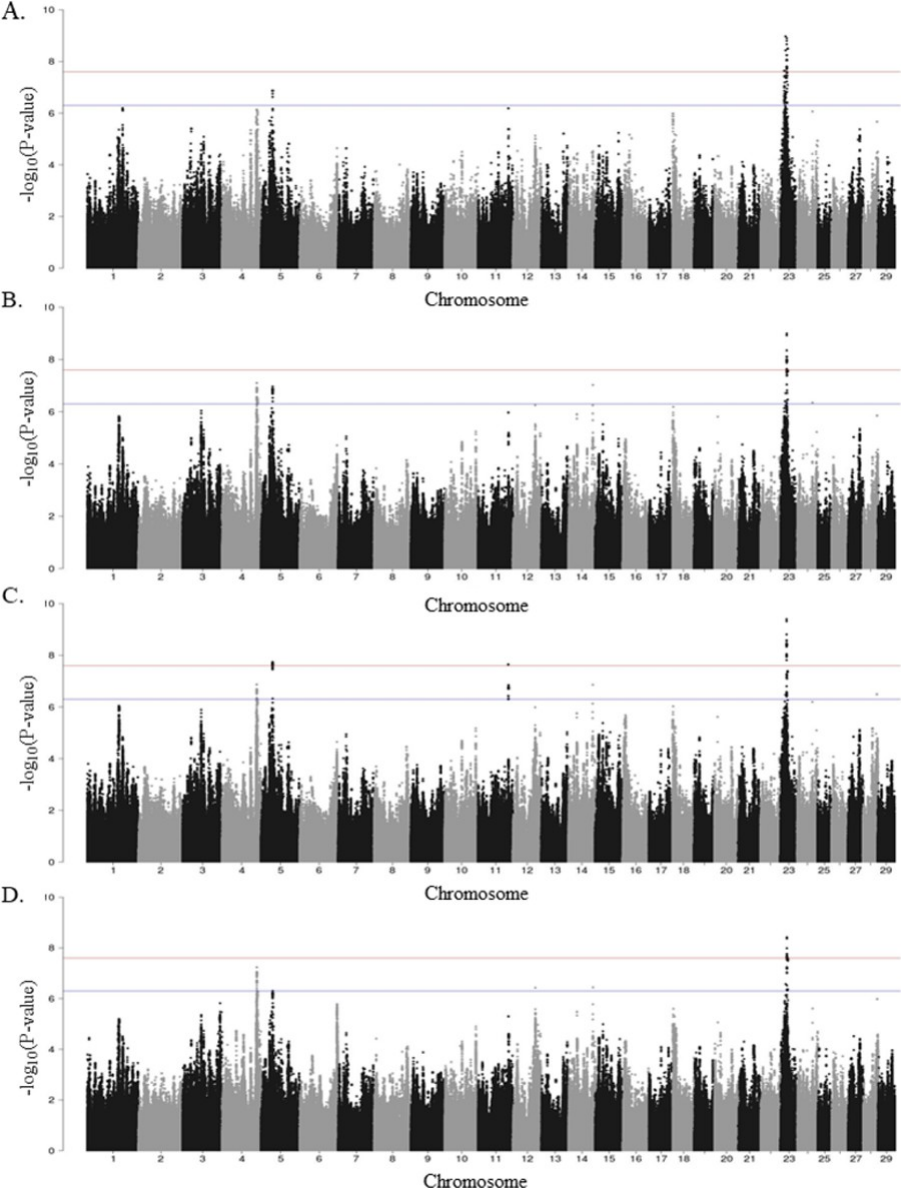
Manhattan plots showing–log_10_(P-values) of association between every single SNP and phenotype. Each dot represents the result from the test association for a single SNP. Animals were considered cases when they were positive to all the tests and controls when they were negative to any (+/), or all of them (+/-). Chromosomes localization of the SNPs associated with ELISA-tissue culture-tissue PCR (+/-) (A), ELISA-tissue culture-tissue PCR (+/) (B), ELISA-tissue culture (+/) (C), and ELISA-tissue PCR (+/) (D) is indicated on the x-axis. The horizontal red line is drawn at–log_10_ (2.5 × 10^−8^), and the horizontal blue line at–log_10_ (5 × 10^−7^) to show the high level of significance.

A total of 3,440 and 159 SNPs were found to be moderate and highly associated with the combination of the ELISA-tissue PCR-tissue culture results (+/-). In this test combination, animals were considered cases when they were positive to all the tests and controls when they were negative to all of them (+/-). Considering the results of the ELISA-tissue culture, the GWAS revealed 4,309 and 238 SNPs moderate, and highly associated with this combination, respectively. As shown in Fig 2 (Manhattan plots), various chromosomal regions were significantly associated with MAP infection according to the combinations of ELISA-tissue PCR-tissue culture (Fig 2A and 2B), ELISA-tissue culture (Fig 2C), and ELISA-tissue PCR (Fig 2D). In all the scenarios, the most significant peak was located on the BTA23, and most of the highly-associated SNPs were located in intronic regions (S1 Fig).

The GWAS defined a total of 12 QTLs highly associated with MAP infection status on 8 chromosomes including BTA4, BTA5, BTA11, BTA12, BTA14, BTA23, BTA24, and BTA28. A description of the SNPs surpassing the threshold (P < 5 × 10^−7^), P-values, along with candidate genes located within the defined QTLs are shown in Table 3. Three QTLs on BTA5 and BTA23 were common to all the test combinations, with the most significant QTLs located on BTA23. Additional highly-associated QTLs were identified on BTA4, BTA11, BTA12, BTA14, BTA23, BTA24, and BTA28. As shown in Table 3, the 159 SNPs associated with the phenotype with the highest h^2^, the ELISA-tissue PCR-tissue culture results (+/-), resided within 5 QTLs which were distributed across BTA5 and BTA23. The BTA23 harbored 4 of the 5 QTL regions. The two QTLs that harbored the most genome-wide significantly-associated SNPs were located on BTA23 (15.09–16.86 and 18.81–22.95 Mb). Using the ELISA-Tissue PCR results, we identified the largest QTL (4.12 Mb) on BTA5. Two QTLs located on BTA11 and BTA28 were specifically associated with the combination ELISA-tissue culture, and one QTL on BTA12 was specifically associated with the ELISA-tissue PCR.

**Table 3:** Quantitative trait loci (QTLs) surpassing the significance threshold (P< 5 × 10^−7^) for evidence of an association with MAP infection status.

Phenotype	BTA^1^	QTL start (bp)	QTL end (bp)	P-value	SNP position^2^	Positional candidate genes in QTL^3^	SNPs in QTL
ELISA Tissue PCR Tissue Culture (+/-)	23	15,060,376	16,864,597	1,06E-09	15,594,386	*NFYA*, *TREM1*, *TREM2*, *TREML1*, *TREML2*, *FOXP4*, *MDFI*, *TFEB*, *PGC*, *FRS6*, *USP49*, *MED20*, *TOMM6*, *BYSL*,*CCND3*, *TAF8*, *GUCA1A*, *MGC137036*, *C23H6orf132*, *GUCA1B*, *MRPS10*, *TERF1*, *U6*, *UBR2*, *PRPH2*, *5S_rRNA*, *TBCC*, *BICRAL*, *RPL7L1*, *PEX6*, *MRPL2SRF*, *PTCRA*, *MEA1*, *CNPY3*, *KLHDC3*, *GNMT*, *RRP36*, *PPP2R5D*, *CUL7*, *KLC4*, *PTK7*, *CUL9*, *SLC22A7*, *DNPH1*, *TTBK1*, *CRIP3*, *ENSBTAG00000021359*, *ENSBTAG00000050887*, *ENSBTAG00000038916*, *ENSBTAG00000054479*, *ENSBTAG00000053124*, *ENSBTAG00000052790*, *ENSBTAG00000051935*, *ENSBTAG00000051409*	13
	23	18,812,221	20,933,699	1,26E-09	19,329,213	*RUNX2*, *U6*, *ENPP4*, *CLIC5*, *ENPP5*, *RCAN2*, *CYP39A1*, *SLC25A27*, *TDRD6*, *ADGRF5*, *PLA2G7*, *ANKRD66*, *ADGRF1*, *TNFRSF21*, *CD2AP*, *ADGRF2*, *ADGRF4*, *OPN5*, *ENSBTAG00000021415*	63
	23	21,898,408	22,958,623	3,19E-09	22,401,117	*MMUT*, *CENPQ*, *GLYATL3*, *C23H6orf141*, *U6*, *RHAG*, *CRISP2*, *CRISP3*, *CRISP1*, *7SK*, *DEFB114*, *DEFB110*, *ENSBTAG00000046237*, *ENSBTAG00000050850*, *ENSBTAG00000053808*, *ENSBTAG00000054396*, *ENSBTAG00000046711*	6
	23	11,332,059	14,196,234	2,32E-08	11,900,592	*CMTR1*, *CCDC167*, *MDGA1*, *ZFAND3*, *BTBD9*, *GLO1*, *DNAH8*, *GLP1R*, *SAYSD1*, *KCNK5*, *KIF6*, *KCNK16*, *KCNK17*, *DAAM2*, *MOCS1*, *U6*, *bta-mir-2377*, *ENSBTAG00000050874*	44
	5	35,035,017	36,111,179	1,35E-07	35,577,195	*ANO6*, *DBX2*, *NELL2*, *TMEM117*, *ENSBTAG00000026249*, *ENSBTAG00000032150*	33
ELISA Tissue PCR Tissue Culture (+/)	23	18,812,221	20,933,699	1,01E-09	19,331,156	*RUNX2*, *U6*, *ENPP4*, *CLIC5*, *ENPP5*, *RCAN2*, *CYP39A1*, *SLC25A27*, *TDRD6*, *ADGRF5*, *PLA2G7*, *ANKRD66*, *ADGRF1*, *TNFRSF21*, *CD2AP*, *ADGRF2*, *ADGRF4*, *OPN5*, *ENSBTAG00000021415*	63
	23	21,898,408	22,958,623	2,61E-08	22,401,993	*MMUT*, *CENPQ*, *GLYATL3*, *C23H6orf141*, *U6*, *RHAG*, *CRISP1*, *CRISP2*, *CRISP3*, *7SK*, *DEFB114*, *DEFB110*, *ENSBTAG00000046237*, *ENSBTAG00000050850*, *ENSBTAG00000053808*, *ENSBTAG00000054396*, *ENSBTAG00000046711*	6
	23	15,094,386	16,094,386	1,95E-07	15,594,386	*TREM1*, *TREM2*, *TREML1*, *TREML2*, *FOXP4*, *MDFI*, *TFEB*, *PGC*, *FRS6*, *USP49*, *MED20*, *TOMM6*, *BYSL*, *CCND3*, *TAF8*, *GUCA1A*, *MGC137036*, *C23H6orf132*, *GUCA1B*, *MRPS10*, *TRERF1*, *ENSBTAG00000050887*, *ENSBTAG00000038916*, *ENSBTAG00000054479*, *ENSBTAG00000053124*	1
	23	13,196,234	14,196,234	3,98E-07	13,696,234	*KIF6*, *KCNK16*, *KCNK17*, *DAAM2*, *MOCS1*, *U6*	1
	4	105,958,204	106,999,289	7,92E-08	106,492,268	*TRBV24-1*, *TRPV6*, *TRBV30*, *TRPV5*, *KEL*, *PRSS2*, *EPHB6*, *OR9A2*, *OR9A16*, *OR9A15*, *OR6V1*, *PIP*, *TAS2R39*, *TAS2R40*, *TMEM139*, *CLCN1*, *GSTK1*, *CASP2*, *FAM131B*, *ZYX*, *EPHA1*, *TAS2R60*, *OR2R3*, *OR10AC1*, *TCAF2*, *TCAF1*, *ENSBTAG00000049004*, *ENSBTAG00000027204*, *ENSBTAG00000050190*, *ENSBTAG00000053376*, *ENSBTAG00000053701*, *ENSBTAG00000053785*, *ENSBTAG00000054917*, *ENSBTAG00000051147*, *ENSBTAG00000047919*, *ENSBTAG00000034609*, *ENSBTAG00000052365*, *ENSBTAG00000050494*, *ENSBTAG00000031162*	93
	4	108,183,737	109,219,627	3,24E-07	108,688,422	* *	10
	5	35,035,017	36,331,608	1,08E-07	35,580,801	*ANO6*, *DBX2*, *NELL2*, *TMEM117*, *5S_rRNA*, *ENSBTAG00000026249*, *ENSBTAG00000032150*	35
	14	74,743,059	75,743,059	9,47E-08	75,243,059	*MMP16*, *CNBD1*, *ENSBTAG00000051556*	1
	24	45,728,397	46,728,397	4,49E-07	46,228,397	*EPG5*, *bta-mir6523a*, *PSTPIP2*, *ATP5F1A*, *HAUS1*, *U6*, *C24H1orf25*, *RNF165*, *LOXHD1*, *ST8SIA5*, *PIAS2*, *KATNAL2*, *ENSBTAG00000048422*, *ENSBTAG00000047360*, *ENSBTAG00000047547*	1
ELISA Tissue Culture (+/)	23	18,812,221	20,933,699	4,01E-10	19,331,156	*RUNX2*, *U6*, *ENPP4*, *CLIC5*, *ENPP5*, *RCAN2*, *CYP39A1*, *SLC25A27*, *TDRD6*, *ADGRF5*, *PLA2G7*, *ANKRD66*, *ADGRF1*, *TNFRSF21*, *CD2AP*, *ADGRF2*, *ADGRF4*, *OPN5*, *ENSBTAG00000021415*	68
	23	21,898,408	22,901,993	4,06E-08	22,401,993	*MMUT*, *CENPQ*, *GLYATL3*, *C23H6orf141*, *U6*, *RHAG*, *CRISP1*, *CRISP2*, *CRISP3*, *7SK*, *DEFB114*, *DEFB110*, *ENSBTAG00000046237*, *ENSBTAG00000050850*, *ENSBTAG00000053808*, *ENSBTAG00000054396*, *ENSBTAG00000046711*	5
	23	15,094,386	16,094,386	3,39E-07	15,594,386	*TREM1*, *TREM2*, *TREML1*, *TREML2*, *FOXP4*, *MDFI*, *TFEB*, *PGC*, *FRS6*,*USP49*, *MED20*, *TOMM6*, *BYSL*, *CCND3*, *TAF8*, *GUCA1A*, *MGC137036*, *C23H6orf132*, *GUCA1B*, *MRPS10*, *TRERF1*, *ENSBTAG00000050887*, *ENSBTAG00000038916*, *ENSBTAG00000054479*, *ENSBTAG00000053124*	1
	11	91,785,241	93,618,888	2,24E-08	92,308,149	*GGTA1*, *DAB2IP*, *NDUFA8*, *MORN5*, *LHX6*, *RBM18*, *MRRF*, *PTGS1*, *bta-mir-10175*, *OR1L18*, *OR1L12*, *OR1L21*,*OR1L8H*, *OR1L8*, *OR1L8C*, *OR1L8E*, *OR1L8D*, *OR1L20*, *OR1N2*, *OR1J26*, *OR1J4E*, *OR1N1*, *OR1J2*, *ENSBTAG00000039201*, *ENSBTAG00000039186*, *ENSBTAG00000012827*, *ENSBTAG00000049150*, *ENSBTAG00000049268*, *ENSBTAG00000048686*, *ENSBTAG00000037739*, *ENSBTAG00000054916*, *ENSBTAG00000055261*, *ENSBTAG00000048884*	43
	5	35,035,017	36,331,608	1,80E-08	35,580,801	*ANO6*, *DBX2*, *NELL2*, *TMEM117*, *5S_rRNA*, *ENSBTAG00000026249*, *ENSBTAG00000032150*	34
	28	42,484,673	43,484,698	3,23E-07	42,984,698	*FRMPD2*, *MAPK8*, *ARHGAP22*, *WDFY4*, *LRRC18*, *VSTM4*, *FAM170B*, *ENSBTAG00000006042*	2
	14	74,743,059	75,743,059	1,38E-07	75,243,059	*MMP16*, *CNBD1*, *ENSBTAG00000051556*	1
	4	105,958,204	106,999,289	1,35E-07	106,492,268	*TRBV24-1*, *TRPV6*, *TRBV30*, *TRPV5*, *KEL*, *PRSS2*, *EPHB6*, *OR9A2*, *OR9A16*, *OR9A15*, *OR6V1*, *PIP*, *TAS2R39*, *TAS2R40*, *TMEM139*, *CLCN1*, *GSTK1*, *CASP2*, *FAM131B*, *ZYX*, *EPHA1*, *TAS2R60*, *OR2R3*, *OR10AC1*, *TCAF2*, *TCAF1*, *ENSBTAG00000049004*, *ENSBTAG00000027204*, *ENSBTAG00000050190*, *ENSBTAG00000053376*, *ENSBTAG00000053701*, *ENSBTAG00000053785*, *ENSBTAG00000054917*, *ENSBTAG00000051147*, *ENSBTAG00000047919*, *ENSBTAG00000034609*, *ENSBTAG00000052365*, *ENSBTAG00000050494*, *ENSBTAG00000031162*	84
ELISA Tissue PCR (+/)	23	18,812,221	22,958,623	3,77E-09	19,331,156	*RUNX2*, *U6*, *ENPP4*, *CLIC5*, *ENPP5*, *RCAN2*, *CYP39A1*, *SLC25A27 TDRD6*, *ADGRF5 PLA2G7*, *ANKRD66*,*ADGRF1*, *TNFRSF21*, *CD2AP*, *ADGRF2*, *ADGRF4*, *OPN5*, *PTCHD*, *OR9G1*, *OR5M10C*, *5S_rRNA*, *MEP1A*, *MMUT*, *CENPQ*, *GLYATL3*, *C23H6orf141*,*U6 RHAG*, *CRISP1*, *CRISP2*, *CRISP3*, *7SK*, *DEFB114*, *DEFB110*, *ENSBTAG00000046237*, *ENSBTAG00000050850*, *ENSBTAG00000053808*, *ENSBTAG00000054396*, *ENSBTAG00000046711*, *ENSBTAG00000021415*, *ENSBTAG00000038677*, *ENSBTAG00000054235*	61
	23	15,094,386	16,094,386	2,66E-07	15,594,386	*TREM1*, *TREM2*, *TREML1*, *TREML2*, *FOXP4*, *MDFI*, *TFEB*, *PGC*, *FRS6*, *USP49*, *MED20*, *TOMM6*, *BYSL*, *CCND3*, *TAF8*, *GUCA1A*, *MGC137036*, *C23H6orf132*, *GUCA1B*, *MRPS10*, *TRERF1*, *ENSBTAG00000050887*, *ENSBTAG00000038916*, *ENSBTAG00000054479*, *ENSBTAG00000053124*	1
	4	105,958,204	107,001,103	5,82E-08	106,492,268	*TRBV24-1*, *TRPV6*, *TRBV30*, *TRPV5*, *KEL*, *PRSS2*, *EPHB6*, *OR9A2*, *OR9A16*, *OR9A15*, *OR6V1*, *PIP*, *TAS2R39*, *TAS2R40*, *TMEM139*, *CLCN1*, *GSTK1*, *CASP2*, *FAM131B*, *ZYX*, *EPHA1*, *TAS2R60*, *OR2R3*, *OR10AC1*, *TCAF2*, *TCAF1*, *ENSBTAG00000049004*, *ENSBTAG00000027204*, *ENSBTAG00000050190*, *ENSBTAG00000053376*, *ENSBTAG00000053701*, *ENSBTAG00000053785*, *ENSBTAG00000054917*, *ENSBTAG00000051147*, *ENSBTAG00000047919*, *ENSBTAG00000034609*, *ENSBTAG00000052365*, *ENSBTAG00000050494*, *ENSBTAG00000031162*	96
	5	35,080,801	36,080,801	4,92E-07	35,580,801	*DBX2*, *NELL2*, *TMEM117*	1
	12	67,715,495	68,715,495	3,73E-07	68,215,495	*GPC6*	1
	14	74,743,059	75,743,059	3,61E-07	75,243,059	*MMP16*, *CNBD1*, *ENSBTAG00000051556*	1

^1^QTL location.

^2^ SNP location in the genome assembly.

^3^ Positional candidate genes are defined as genes that are located within 50 kb on either side of the identified QTL. When combinations of diagnostics tests were used, animals were considered cases when they were positive to all the tests and controls when they were negative to any (+/) or all of them (+/-).

Quantile-quantile plots comparing the observed distribution of–log (P-values) to the expected values under null hypothesis are shown in S2 Fig. The plots showed a distribution close to the expected distribution line for the following phenotypes: ELISA-tissue PCR-tissue culture (+/-) (λ_median_ = 1.007), ELISA-tissue PCR-tissue culture (+/) (λ_median_ = 1.004), ELISA-tissue culture (+/) (λ_median_ = 1.006), and ELISA-tissue PCR (+/) (λ_median_ = 1.006), indicating that significant values were not overestimated due to population stratification or cryptic relatedness. A slight deviation in the upper right tails from the y = x line suggested that some association was present in the data.

Table 3 also presents a list of candidate genes either containing highly-associated SNPs or within 50 kb of the surrounding genomic region of the highly-associated SNPs. We were able to identify candidate genes in all the QTLs except for one located in BTA4. One hundred and fourteen candidate genes were mapped within the 5 QTLs associated with the phenotype with the highest h^2^, the ELISA-tissue culture-tissue PCR (+/-), the majority located on BTA23. The OR calculated for all the SNPs associated with a positive ELISA-tissue PCR-tissue culture diagnosis (+/-) (P < 5 × 10^−7^) were > 1 indicating that the animals with the minor alleles were more likely to have a positive ELISA-tissue PCR-tissue culture result. The OR for the SNPs defining the peak of each QTL are shown in Table 4 under the codominant genetic model.

**Table 4:** Odds ratio (OR) for some the SNPs associated with a positive ELISA-tissue PCR-tissue culture diagnosis (+/-)(P < 5 x 10^−7^).

Phenotype	BTA	SNP position	SNP ID	Genotype	Cases (%)	Controls (%)	OR (95% CI)
ELISA Tissue PCR Tissue Culture (+/-)	23	15,594,386		CCATGGGGT/CCATGGGGT	29 (52.7)	566 (86.0)	1.0
				C/CCATGGGGT	24 (43.6)	89 (13.5)	4.7 (2.6–8.5)
				C/C	2 (3.6)	3 (0.5)	17.5 (2.6–118.3)
	23	19,329,213	rs209106537	C/C	20 (36.4)	461 (70.1)	1.0
				A/C	25 (45.5)	181 (27.5)	3.2 (1.7–5.9)
				A/A	10 (18.2)	16 (2.4)	13.2 (5.2–33.2)
	23	22,401,117	rs385614395	G/G	33 (60.0)	578 (87.8)	1.0
				C/G	18 (32.7)	78 (11.9)	3.8 (2.0–7.2)
				C/C	4 (7.3)	2 (0.3)	43.3 (7.5–249.9)
	23	11,900,592	rs136621504	G/G	37 (67.3)	607 (92.2)	1.0
				A/G	18 (32.7)	51 (7.8)	5.5 (2.9–10.4)
	5	35,577,195	rs110029938	T/T	36 (65.5)	583 (89.1)	1.0
				C/T	17 (30.9)	71 (10.8)	4.2 (2.2–8.0)
				C/C	2 (3.6)	1 (0.2)	44.1 (3.6–538.5)

(+/-) Animals were considered cases when they were positive to all the tests and controls when they were negative to all of them.

## Discussion

Previous studies that estimated genetic parameters for PTB in cattle indicated that susceptibility to MAP infection is heritable, with h^2^ estimates of susceptibility to the disease ranging from < 0.01 (16) to 0.2843 (18). This is comparable with the moderate heritability (h^2^ = 0.12) estimated for bTb infection even though the phenotype definitions and models used differed [42]. In our study population, the h^2^ estimates were also moderate (ranged from 0.054 to 0.139). Interestingly, we observed that the combination of multiple diagnostic tests increased the h^2^ estimates, with the highest h^2^ obtained for the combination of ELISA-tissue PCR-tissue culture (+/-) (h^2^ = 0.139). For this phenotype, all the common SNPs explained ~14% of the phenotypic variance. Except for the tissue culture (h^2^ = 0.101), the low h^2^ estimates obtained for the individual tests (h^2^ < 0.08) could be attributed to their lack of sensitivity for the detection of animals that are in the subclinical stages of MAP infection (false negatives). While cows testing ELISA-tissue PCR and tissue culture positive are very likely infected, animals could be test-negative because of a false-negative test result, lack of exposure to MAP, or be truly resistant. While a positive test result is almost certainly associated with disease susceptibility, a negative test does not always reflect resistance. In agreement with this, we observed that all the SNPs associated with the ELISA-tissue PCR-tissue culture diagnosis (+/-) had an OR > 1 and were, therefore, associated with disease susceptibility. This implies that susceptibility to MAP infection can be modified using genetic selection. In our study population, only 9 of the 70 ELISA-positive animals couldn´t be detected by tissue PCR and culture. As above, these cows could be tissue PCR and culture negative because of a false-negative test result or be truly resistant. PTB resistance defined as the ability of the host to clear the pathogen by mounting a protective immune response deserves further studies.

Our study is unique in the definition of cases and controls through the combination of multiple diagnostic tests including ELISA for detection of humoral responses against MAP, and culture and PCR detection of MAP in tissue samples. Moreover, the population size used (N = 983) was bigger than in a previous GWAS using the bovine SNP50 Bead Chip where cases were defined as tissue PCR-tissue culture positives (N = 459) [30]. Although a previous study combined the data from two GWAS to identify loci associated with MAP tissue infection and humoral immune response [20], our study provides the first comparison of the genetic effects associated with different phenotypic measurements or diagnostic definitions in a common set of samples using WGS data. Combination test interpretation (all tests negative equals non-infected and all tests positive equals infected) was used to increase the sensitivity of the ELISA-Tissue PCR-tissue culture combination. In addition, the use of imputed WGS increased the accuracy of the GWAS enabling the identification of 12 QTLs surpassing the high threshold (P < 5 × 10^−7^) for evidence of an association with MAP infection. These strategies increased the hereditability of the trait and reduced the risk of misclassification. Further studies will demonstrate whether the post-mortem examination of gut tissues and regional lymph nodes can improve, even more, the accuracy of the classification of naturally infected animals and uninfected controls, and provides high h^2^ estimates. Once a PTB-associated phenotype with a moderate/high h^2^ estimate is finally selected, genomic predictions will be developed in small, well-recorded reference populations using the genomic BLUP model, and then the prediction equation will be applied to predict genomic estimated breeding values of Holstein cattle that were genotyped but did not have PTB-associated phenotypes themselves.

Identifying significant SNPs and QTLs associated with PTB susceptibility is extremely important to understand the molecular mechanisms involved in the pathogenesis of the disease. Our results confirmed that susceptibility to PTB is polygenic with a large number of genetic variants each having a small effect on the regulation of the observed phenotype. Using combinations of diagnostic tests and genotypes imputed to the WGS level, we identified a total of 12 regions associated with PTB susceptibility (P < 5 x 10^−7^) on 8 chromosomes (BTA4, BTA5, BTA11, BTA12, BTA14, BTA23, BTA24, and BTA28). By examining the available cattle QTL database, we observed that the identified QTLs on BTA23 (18.81–22.95 Mb), BTA24 (45.72–46.72 Mb), and BTA12 overlapped with four regions previously associated with PTB susceptibility; QTL14876, QTL166685, QTL139831, and QTL211947 [25,29,32,43]. This finding provided additional evidence that genes within these regions such as the *adhesion G protein-coupled receptor F1* (*ADGRF1)*, *adhesion G protein-coupled receptor F5 (ADGRF5)*, *TNF receptor superfamily member 21* (*TNFRSF21)*, *defensin β110* (*DEFB110*) and *defensin β114* (*DEFB114*) are likely associated with MAP infection. Previous studies supported a role for the β-defensins as important host defense effector molecules that are rapidly mobilized by the epithelium upon MAP infection [44]. *ADGRF1*and *ADGRF5*, also named *GPR110* and *GRP116*, are G-protein-coupled receptors acting as important regulators in the progression and development of several human inflammatory diseases including IBD, hepatocellular carcinoma, lung cancer, gastric cancer, prostatic cancer, glioma, and colorectal cancer [45]. The *TNFRSF21* is a member of the *TNF/TNFR* family and plays a critical role in pathogen recognition, immune response, inflammation, and tumor progression [46,47]. The encoded protein by this gene activates *nuclear factor kappa-B* (*NF-κβ*) and *mitogen-activated protein kinase 8* (*MAPK-8*, also called c*-Jun N-terminal kinase 1*) and induces cell apoptosis. Fang et al. demonstrated that the level of *TNFRSF21* was dramatically increased in bovine peripheral blood leucocytes of mastitis cows, which underlines its important regulatory role in bovine mastitis inflammation [48]. More recently, a role for the *TNFRSF21* in the regulation of bovine mastitis susceptibility via GWAS and post-transcriptional analysis was discovered [49]. Interestingly, several PTB-associated QTLs identified in the current study were located on regions previously found to be associated with clinical mastitis including QTL30823, QTL30824, QTL30825, QTL30826, QTL161601, QTL161607, and QTL65863 [48–52].

Other candidate genes identified in BTA23 (18.81–22.95 Mb) include genes involved in the splicing process (*U6*), invasion and migration of tumors (*CLIC5*, *ENPP4*, *ENPP5*), and in the regulation of the *calcineurin-nuclear factor of activated T cells* (*RCAN2*). This region overlapped with QTLs on BTA23 associated with bTb infection in a multi-breed GWAS of Charolais, Limousin, and Holstein Friesian (genetic positions 19.44–22.72 Mb) [42]. Interestingly, this region included the *RCAN2* gene as well. The *RCAN2* is responsible for the regulation of *calcineurin 2*, and *calcineurin* activation promotes the survival of *Mycobacterium tuberculosis* (MTb) within its host by preventing phagocyte maturation which is required to destroy intracellular bacteria [53]. Our results, together with previous studies, suggested that regulation of *calcineurin 2* may have a pivotal role in the susceptibility of cattle to both bTb and PTB. In addition, a second PTB-associated QTL on BTA23 (11.32–14.19 Mb) overlapped with a bTb-associated QTL (QTL96552) [54].

Several positional candidate genes that we identified in other QTLs were important transcriptional regulators, including the *Forkhead BoxP4 (FOXP4*), *transcription factor EB* (*TFEB)*, the *LIM/homeoboxprotein* (*LHX6)*, and the mediator of RNA polymerase II transcription subunit 20 (*MED2*). The *TFEB* acts as a master regulator of lysosomal biogenesis, autophagy, lysosomal exocytosis, lipid catabolism, energy metabolism, and immune response against intracellular pathogens including MTb [55]. Autophagy, an intracellular lysosomal degradation process, is an important cell-autonomous defense system involved in innate and adaptive immune responses and contributes to host defense against various intracellular microbes. Among the transcription factors implicated in autophagy, *TFEB* is a critical regulator of autophagic activation. In the current study, several candidate genes potentially associated with MAP infection were involved in the splicing process, including the U6 spliceosomal RNA and 7SKRNA. Further studies are needed to determine if the identified SNPs are affecting the positional candidate genes or other genes through cis- or trans-regulatory effects [56].

The candidate genes identified in this study are novel in the sense that they have not been associated with PTB risk in cattle before. Interestingly, some of the candidate genes identified in the current study were previously found associated with mastitis and bTb infection. For instance, the *RCAN2* gene was found associated with bTb infection [42] and with 18 other bovine traits including somatic cell counts [57]. Although the *TNFRSF21* has not been associated with PTB risk in cattle, other members of the *TNF* receptor superfamily such as *TNFRSF18* and *TNFRSF4* are known to stimulate the Th1 cell-mediated immune response and have been previously identified as PTB candidate genes [28]. As mentioned before, the *TNFRSF21* was identified to be involved in the regulation of bovine mastitis susceptibility via GWAS [49].

The candidate genes identified in our study were also compared to candidate genes that were previously identified in CD, IBD, and colorectal cancer to determine if there was any overlap. Five of the candidate genes identified in the current study; the *TNFRSF21*, *ADGRF1*, *FOXP4*, *Cyclin D3 (CCND3*), and *Transmembrane Protein* 117 (*TMEM117*) were previously found to be associated with IBD [58]. *FOXP4* and *CCND3* were also found associated with CD. Recent association analysis identified a new risk locus harboring the *TFEB* for colorectal cancer susceptibility [59]. Using the candidate genes identified in our study, we could not find any enriched pathway underlying PTB susceptibility. However, several of the identified candidate genes matched genes in the Innate DB database with relevant innate immunity functions such as the t*riggering Receptor Expressed On Myeloid Cells 1 and 2 (TREM1*, *TREM2)*, t*riggering Receptor Expressed On Myeloid Cells like 1 and 2 (TREML1*, *TREML2)*, *Canopy FGF Signaling Regulator 3 (CNPY3)*, Protein *Phosphatase 2 Regulatory Subunit B’Delta (PPP2R5D)*, *Cysteine Rich Secretory Protein 3 (CRISP3)*, *Anoctamin 6 (ANO6)*, *Protein Inhibitor of Activated STAT2 (PIAS2)*, *DAB2 Interacting protein (DAB2IP)*, and *MAPK8*.

## Conclusions

In summary, combining phenotypes and WGS into a joint GWAS improved the power for detecting genetic associations in Spanish Holstein cattle. Our results validated some previously reported associations and identified novel SNPs, QTLs, and candidate genes associated with the antibody response to MAP infection and with MAP detection in infected tissues. Furthermore, the addition to genotyping assays of the SNPs identified in the current study would allow producers to select cattle that are less susceptible to PTB and likely to other bovine diseases as well; ultimately reducing the spread of diseases, preventing further economic losses, and reducing antimicrobial use. Consequently, a reduction of the presence of MAP in the environment may also be beneficial to humans, especially if the link between MAP and human inflammatory diseases is confirmed.

## Supporting information

S1 Fig Genomic distribution of the SNPs surpassing the significance threshold (P < 5 × 10^−7^) for evidence of an association with MAP infection status. The chart depicts the genomic distribution of the SNPs associated with (A) ELISA-tissue culture-tissue PCR (+/-), (B) ELISA-tissue culture-tissue PCR (+/), (C) ELISA-tissue culture (+/), and (D) ELISA-tissue PCR (+/) according to the Ensembl Variant Effect Predictor (VEP).
(TIF)

S2 Fig Quantile-quantile plots of–log (P-values) for genome-wide association analysis for susceptibility to MAP infection. The plots showed a distribution close to the expected distribution line for the following phenotypes: ELISA-tissue PCR-tissue culture (+/-) (λ_median_ = 1.007), ELISA- tissue PCR-tissue culture (+/) (λ_median_ = 1.004), ELISA-tissue culture (+/) (λ_median_ = 1.006), and ELISA-tissue PCR (+/) (λ_median_ = 1.006). The red line is the slope expected under no inflation and no true association, the y-axis represents the observed–log (P-values), and the x-axis represents the expected–log (P-values), under the null hypothesis of no association.
(TIF)
