# Supplementary figures and images for: Identification of loci associated with susceptibility to Mycobacterium avium subsp. paratuberculosis infection in Holstein cattle using combinations of diagnostic tests and imputed whole-genome sequence data
Source: PLoS One. 2021 Aug 27;16(8):e0256091. doi: 10.1371/journal.pone.0256091 (PMC8396740; doi:10.1371/journal.pone.0256091)

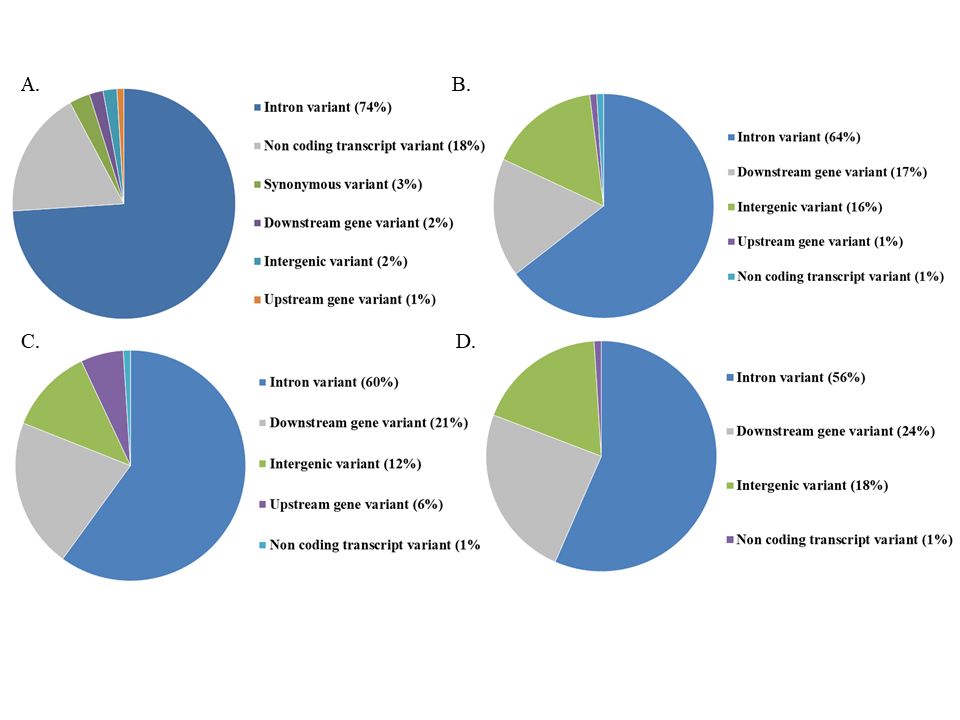

Supplement: S1 Fig — The chart depicts the genomic distribution of the SNPs associated with (A) ELISA-tissue culture-tissue PCR (+/-), (B) ELISA-tissue culture-tissue PCR (+/), (C) ELISA-tissue culture (+/), and (D) ELISA-tissue PCR (+/) according to the Ensembl Variant Effect Predictor (VEP). (TIF) [file pone.0256091.s001.tif]

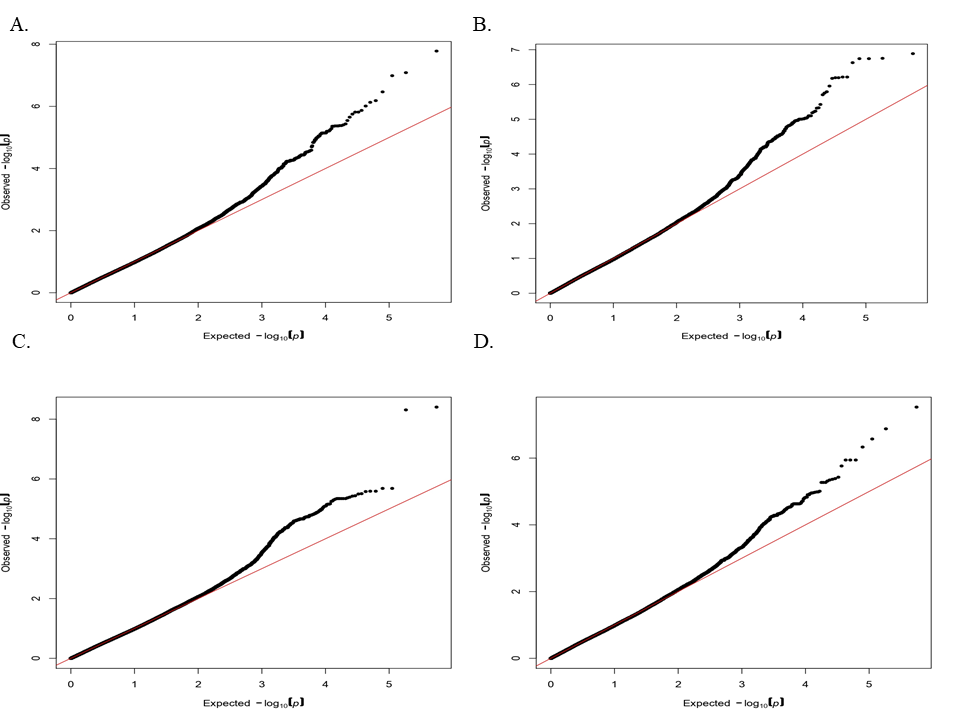

Supplement: S2 Fig — The plots showed a distribution close to the expected distribution line for the following phenotypes: ELISA-tissue PCR-tissue culture (+/-) (λmedian = 1.007), ELISA- tissue PCR-tissue culture (+/) (λmedian = 1.004), ELISA-tissue culture (+/) (λmedian = 1.006), and ELISA-tissue PCR (+/) (λmedian = 1.006). The red line is the slope expected under no inflation and no true association, the y-axis represents the observed–log (P-values), and the x-axis represents the expected–log (P-values), under the null hypothesis of no association. (TIF) [file pone.0256091.s002.tif]
